# An Uncommon Case of Recurrent Hypoglycemic Episodes in a Healthy Non-diabetic Male: Insulin Autoimmune Syndrome

**DOI:** 10.7759/cureus.41183

**Published:** 2023-06-30

**Authors:** Kanwarpal K Dhaliwal, Gaurav Bector, Saurabh Arora, Amanpreet Singh, Sanjay Kalra

**Affiliations:** 1 Medicine and Surgery, Dayanand Medical College and Hospital, Ludhiana, IND; 2 Endocrinology, Dayanand Medical College and Hospital, Ludhiana, IND; 3 Endocrinology, Bharti Hospital, Karnal, IND

**Keywords:** secretagogues, hyperinsulinemic hypoglycemia, hypoglycemia, insulin autoantibodies, insulin autoimmune syndrome

## Abstract

Insulin autoimmune syndrome is a rare cause of recurrent hypoglycemic episodes that can mimic various other pathological problems leading to unnecessary diagnostic assessments and interventions. Here, we report a case of a healthy non-diabetic male in his 50s presenting with recurrent episodes of hypoglycemia with no prior exposure to exogenous insulin. During a 72-hour fasting test, his glucose levels reached 22 mg/dl within less than three hours. The lab tests showed insulin of 1000 μIU/mL and C-peptide of 4.99 ng/ml. On further evaluation, high titers of insulin autoantibodies (IAA) >100 U/ml (normal = <10 U/ml) were consistent with insulin autoimmune syndrome diagnosis. This case thus highlights the importance of including IAA titers in first-line investigations for hypoglycemia in a non-diabetic patient with strikingly high blood insulin levels.

## Introduction

Hypoglycemia is not a very common clinical event except in those who use hypoglycemic agents for the treatment of diabetes mellitus. In this case, we present a healthy non-diabetic male with recurrent episodes of hyperinsulinemic hypoglycemia with no history of prior exposure to exogenous insulin or secretagogues. Out of all the causes of endogenous hyperinsulinism, insulin autoimmune syndrome is rare in ethnicities other than Japanese. Insulin autoimmune syndrome is characterized by hyperinsulinemic hypoglycemia with raised insulin autoantibodies (IAA) titers, absence of pathological abnormality of the pancreas, and no history of any prior exposure to exogenous insulin. It is noteworthy that insulin autoimmune syndrome can mimic several other pathological scenarios leading to unnecessary diagnostic assessments and interventions. Moreover, it is difficult to differentiate insulin autoimmune syndrome from other forms of endogenous hyperinsulinemic hypoglycemia without measuring IAA titers. This case report thus emphasizes the importance of testing for IAA in non-diabetic adults with hyperinsulinemic hypoglycemia.

## Case presentation

A non-diabetic Indian man in his 50s presented to our clinic with recurrent episodes of hypoglycemia for the past two weeks. These episodes occurred in fasting and postprandial states and were characterized by neurogenic and neuroglycopenic symptoms, including sweating, anxiety, and behavioral changes. The symptoms were corrected by consuming carbohydrate-based food. He denied the use of any hypoglycemic agents, insulin, or dietary supplements containing alpha-lipoic acid. There was no history of intake of drugs containing sulfur or sulfhydryl group. Other than chronic obstructive pulmonary disease and beta-thalassemia minor, there was no history of autoimmune disease or concomitant or recurrent viral infections. Family history was also insignificant except for positive beta-thalassemia minor in the father. There was no history of significant weight loss. He was on ipratropium bromide, salbutamol, and montelukast for chronic pulmonary obstructive disease.

Investigations

The patient was admitted for a 72-hour fasting test, and less than three hours later, he began to experience hypoglycemic symptoms. The lab tests showed fasting blood glucose of 22 mg/dl, insulin of 1000 micro IU/mL, C-peptide of 4.99 ng/ml, and cortisol of 13.46 microgram/dl. To rule out an insulinoma, a contrast-enhanced computed tomography (CECT) of the entire abdomen was performed. As a result, we looked for a less common reason and measured IAA, insulin receptor antibodies, and other autoimmune indicators. High IAA titers were observed (>100 U/ml, normal = <10 U/ml). The anti-nuclear antibody profile, however, was found to be inactive. The patient was started on continuous dextrose infusion given recurrent episodes of hypoglycemia. All the requirements were satisfied, leading to the diagnosis of insulin autoimmune syndrome: hyperinsulinemic hypoglycemia, high titers of IAA, absence of anatomical pancreatic abnormalities, and lack of prior exogenous insulin administration. Other than this, the complete blood count showed microcytic hypochromic anemia in the setting of mild anemia (hemoglobin = 11.2g/dl), normal serum iron levels, and a normal erythrocyte count with a positive family history of thalassemia in the father, suggesting beta-thalassemia minor. Hemoglobin electrophoresis was done to corroborate the results, which showed decreased hemoglobin A (HbA), higher hemoglobin A2 (HbA2) levels, and a slight increase in hemoglobin F (HbF) levels. The erythrocyte sedimentation rate (ESR) was raised to 60 mm/hour (normal = 0-20 mm/hour). Serum protein electrophoresis showed no evidence of monoclonal gammopathy. The routine urine test and liver and renal function tests were insignificant. Tests to measure thyroid function were within normal bounds.

Treatment

The patient was managed with a 10% intravenous dextrose infusion drip and prednisolone (1 mg/kg/day). Montelukast was discontinued. Levels of blood glucose and electrolytes were constantly monitored. The dextrose drip was tapered slowly over three days, after which the patient did not have repeat episodes of hypoglycemia and was discharged in an asymptomatic condition. The dose of prednisolone was gradually decreased to 10 mg/day over two months. The patient remained asymptomatic and monitoring of blood glucose levels at home did not reveal any episode of hypoglycemia.

## Discussion

Our patient presented with an episode of hypoglycemia in both fasting and postprandial states. On evaluation, strikingly high insulin levels during a hypoglycemic episode without any abnormality on abdominal imaging prompted us to search for autoimmune etiology. Elevated levels of IAA confirmed the diagnosis of insulin autoimmune syndrome. Prompt response to steroids further favored the diagnosis. The patient was on inhaled ipratropium bromide plus salbutamol and montelukast, out of which the latter was discontinued. Therefore, the possibility of montelukast-induced autoimmune hypoglycemia cannot be ruled out at this moment.

Insulin autoimmune syndrome is a rare cause of hyperinsulinemic hypoglycemia with raised IAA titers and absence of pathological abnormality of the pancreas with no history of any prior exposure to exogenous insulin. In Japan [1], it is the third leading cause of spontaneous hypoglycemia after insulinoma and extra-pancreatic neoplasia. However, it is rare in other ethnicities. Interestingly, up to 2020, only 28 cases have been reported from India [2], the second most populated country in the world. But its exact incidence is a matter of debate as it has been probably underestimated due to general unawareness of the disease, its transient nature, and difficulties in the diagnostic workup.

Patients with insulin autoimmune syndrome usually present in adulthood with postprandial hypoglycemia, which was consistent with its pathophysiology. However, fasting hypoglycemia or even unpredictable hypoglycemic episodes have also been reported. A strong association has been observed [3] between insulin autoimmune syndrome and the presence of human leukocyte antigen (HLA)-DR4 and HLA-DRB1*0406. Although the syndrome's pathogenesis has not been fully understood, the most widely accepted hypothesis [2] is that the binding and subsequent release of secreted insulin by autoantibodies leads to a mismatch between blood glucose and free insulin concentration. Following the consumption of a meal, the blood glucose level rises to provide a stimulus for insulin secretion. The autoantibodies bind to these insulin molecules making them ineffective. The resultant hyperglycemia thus promotes further insulin production. Once the binding capacity of IAA is exceeded and the free insulin normalizes the glucose levels, pancreatic insulin secretion stops. However, increased insulin reserves due to the formation of autoantibody-insulin complexes and its subsequent release from antibodies lead to hypoglycemia. The severity and duration of resultant hypoglycemia depend on the intrinsic dissociation rate constant, antibody titer, and its affinity/avidity to insulin. Medications or their metabolites containing sulfhydryl or sulfur groups [3] may be able to bind and reduce the sulfhydryl bonds connecting insulin chains A and B, making endogenous insulin more immunogenic. Viruses may act as superantigens triggering the production of autoantibodies [3] and thereby causing insulin autoimmune syndrome. Association with other autoimmune and hematological diseases [3] has also been reported.

A striking feature of insulin autoimmune syndrome is the magnitude of insulin elevation, with results generally above 1000 pmol/L. This is due to the increased half-life of autoantibody-bound insulin. However, the levels of C-peptide and proinsulin vary [3], depending on the capability of the antibody to bind to either of them as well as on its ability to interfere with the immunoassay used. Therefore, insulin autoimmune syndrome can mimic several pathological scenarios [4], prompting expensive and useless diagnostic assessments and interventions. The Endocrine Society thus has emphasized the importance of testing for IAA in non-diabetic adults with hyperinsulinemic hypoglycemia by including it among the first-line tests [5], performed in such patients. Moreover, the results of a Korean study [6] also prove that insulin autoimmune syndrome and other forms of endogenous hyperinsulinemic hypoglycemia cannot be differentiated without measuring IAA titers. It is also noteworthy that recurrent hypoglycemia lowers the glycemic threshold for cortisol secretion. Therefore, a seemingly low plasma cortisol concentration during hypoglycemia does not necessarily indicate adrenocortical insufficiency [5] in the absence of other clinical clues.

In many patients, insulin autoimmune syndrome is self-limiting with spontaneous resolution within three to six months after stopping the trigger drug. For those with intractable hypoglycemia, small frequent meals [3] and low in carbohydrates remain the first line of treatment. It prevents fasting and avoids glucose overload leading to postprandial hyperglycemia and then hypoglycemia. Continuous glucose monitoring helps to predict and respond promptly to falling blood glucose levels in severe cases. As this is an autoimmune-based disease, it has also been treated with corticosteroids with good results. In more severe cases, plasmapheresis can be used alone [1], especially when corticosteroids are contraindicated [3], or in combination with corticosteroids. Recently, rituximab [1] has been introduced for the treatment of patients with life-threatening hypoglycemia and refractory to high-dose corticosteroids. Acarbose, an α-glucosidase inhibitor, is another option for treatment [3], as it dampens the postprandial rise in serum glucose and insulin. But it is poorly tolerated due to its gastrointestinal adverse effects.

## Conclusions

Documented recurrent episodes of symptomatic hypoglycemia in patients with severe adrenergic, cholinergic, and neuroglycopenic features often put the treating physician in a dilemma regarding disease pathophysiology, diagnosis, and treatment. Therefore, it is necessary to address these learning objectives. Insulin autoimmune syndrome remains a strong differential in patients with insulin levels >100 micro IU/mL. The presence of increased levels of IAA is required to distinguish between the insulin autoimmune syndrome and other causes of endogenous hyperinsulinemic hypoglycemia. Discontinuation of the offending drug, dietary modifications, and glucocorticoids remain the treatment of choice.
